# Study on the Application Effect of Fast Track Surgery Care Combined With Continuous Care After Discharge in Patients With Laparoscopic Cholecystectomy

**DOI:** 10.3389/fsurg.2022.848234

**Published:** 2022-02-21

**Authors:** Jian Yu, Xiao Lin, Hong Chen

**Affiliations:** Operating Room, The First People's Hospital of Lianyungang, Jiangsu, China

**Keywords:** laparoscopic cholecystectomy, fast track surgery care, continuous care after discharge, surgical stress levels, Hamilton anxiety scale

## Abstract

**Purpose:**

To explore the application effect of fast track surgery (FTS) care combined with continuous care after discharge in patients with laparoscopic cholecystectomy (LC).

**Methods:**

Two hundred patients treated with LC in our hospital from May 2020 to September 2021 were selected and divided into the routine group receiving routine care (*n* = 100) and the combined group receiving FTS care combined with continuous care after discharge (*n* = 100) according to their care methods. We observed the care effect, surgical stress levels [epinephrine, cortisol, Hamilton anxiety scale (HAMA)], postoperative recovery (time to first exhaust, time to first meal, time to first getting out of bed, time to hospitalization), complications, SF-36 scores after discharge, and care satisfaction in both groups.

**Results:**

The total efficiency of care in the combined group was better than that in the routine group (*P* < 0.05). At 1 d after surgery, the levels of epinephrine and cortisol in both groups were significantly higher than those at 1 h before surgery, and the HAMA scores were significantly lower than those at 1 h before surgery, and the combined group was lower than the routine group (*P* < 0.05). The time to first exhaustion, time to first meal, time to first getting out of bed, and time to hospitalization were shorter in the combined group than in the routine group (*P* < 0.05). The overall complication rate in the combined group was lower than that in the routine group (*P* < 0.05). The each item of SF-36 scores after discharge were higher in the combined group than in the routine group (*P* < 0.05). The total satisfaction with care was higher in the combined group than in the routine group (*P* < 0.05).

**Conclusion:**

The implementation of FTS care combined with continuous care after discharge in LC patients is ideal, which can significantly reduce the level of surgical stress, accelerate the recovery process, and reduce the occurrence of complications, and improve the postoperative quality of life of patients significantly, and with high satisfaction, which is worthy of application.

## Introduction

With the improvement of material living standard and the change of diet structure, the prevalence of gallbladder disease in China is increasing year by year. The disease mainly presents with symptoms such as epigastric pain and abnormal liver function (1). At the beginning of its onset, the patient's symptoms are often insignificant, but as the disease progresses, it may cause shock and threaten the patient's life and health (2). For the intervention of gallbladder disease, the clinical treatment of surgical resection of lesions can benefit every patient, but the perioperative stress associated with surgery inevitably affects the patient's recovery and is closely related to postoperative complications (3, 4). How to effectively control surgery-related stress and reduce postoperative complications to accelerate patient recovery has become a hot topic of current research. Laparoscopic surgery is a minimally invasive technique in which a specially designed catheter is inserted into the patient's peritoneal cavity and passed through the laparoscope to expand the view of the operative area and thus remove the lesion more completely (5). Its application to focal resection in gallbladder disease has been shown to significantly reduce intraoperative blood loss and reduce surgical stress (6, 7). At the same time, the fast track surgery (FTS) care concept suggests that the blocking of stress in surgical patients should include preoperative care of the patient's physical and mental health, care to reduce the stress of therapeutic measures, and care to block afferent nerve conduction stress signals (8, 9). Once the concept was introduced, it was quickly adopted as a new model of perioperative care in many countries in Europe and the United States. In this study, FTS care was applied to patients undergoing laparoscopic cholecystectomy (LC), and it is not known whether the combination of the two has a superimposed effect on accelerating the patients' postoperative recovery. In addition, it has been suggested that the lack of continuity and compactness of clinical care for patients by health care professionals under the conventional care model makes it difficult for patients to obtain adequate professional care after discharge, which is not conducive to the advantages of LC treatment (10). Based on the above, in order to improve the quality of care services in our hospital, enhance the outcome of LC surgical treatment and improve the prognosis of patients, in recent years, our department has seamlessly connected FTS care with continuous care after discharge and observed the effect of its application to LC patients.

## Materials and Methods

### Research Object

Two hundred patients treated with LC in our hospital from May 2020 to September 2021 were selected. Inclusion criteria: Meeting the indications for LC, i.e., symptomatic gallbladder stones, symptomatic chronic cholecystitis, symptomatic and surgically indicated gallbladder augmentation disease (11); Those who were cognitively normal and could cooperate with the relevant questionnaire assessment; Those who had signed the informed consent form. Exclusion criteria: Those treated with combined choledochoscopy or duodenoscopy; Those with intraoperative conversion to open surgery; Those with gallbladder stones combined with biliary stones; Those with illiteracy or combined cognitive impairment or cerebrovascular pathology; Those with combined diabetes mellitus or severe cardiopulmonary disease; Those with combined malignancy; Those with contraindications to LC, such as acute cholecystitis with severe complications, obstructive jaundice, gallbladder augmentation disease suspected to be cancerous, peritonitis, etc. (12); Those with combined bleeding disorders or coagulation disorders. They were divided into the routine group receiving routine care (*n* = 100) and the combined group receiving FTS care combined with continuous care after discharge (*n* = 100) according to the method of care. Comparing the general conditions such as age and primary illness between the two groups was not statistically significant and could be used for comparative studies (*P* > 0.05) (Table 1).

**Table 1:** Comparison of general conditions of two groups (*n*, $\bar{x}$±s, %).

**Itmes**	**Routine group (*n* = 100)**	**Combined group (*n* = 100)**	**t/χ^2^ value**	***P-*value**
Age (years old)	40.93 ± 5.28	42.05 ± 5.16	1.517	0.131
BMI (kg/m^2^)	24.32 ± 2.04	24.65 ± 2.11	1.124	0.262
Gender (cases)			0.188	0.664
Male	62 (62.00)	59 (59.00)		
Female	38 (38.00)	41 (41.00)		
Primary disease (cases)			0.239	0.887
Gallbladder stones	55 (55.00)	57 (57.00)		
Gallbladder polyps	27 (27.00)	24 (24.00)		
Others	18 (18.00)	19 (19.00)		

### Research Methods

The routine group received routine care: (1) preoperative medical staff informed fasting for 6 h, no drinking for 2 h, surgical and anesthetic precautions, and postoperative functional exercises methods; (2) preoperative routine placement of gastric tube (removed after anal venting) and urinary catheter (removed 3 ~ 5 days after surgery); (3) preoperative evening clean enema; (4) half an hour before surgery, intramuscular injection of atropine 0.5 mg + midazolam 3 mg in the ward; (5) intraoperative routine general anesthesia care; (6) postoperative drainage tubes were routinely left in place and removed for 24 ~ 48 h; (7) pain pumps were not routinely used, and opioids were used for pain relief when necessary; (8) eating and watering after the anus was exhausted after surgery; (9) patients got out of bed when voluntary; (10) fluid control was 2,500 ~ 3,000 ml on the postoperative day.

The combined group received FTS care combined with continuous care after discharge care. FTS care: (1) preoperative medical staff informed fasting for 6 h, no drinking for 2 h, adopting diversified forms of health education (information, pictures, electro-educational films, etc.) to popularize knowledge, precautions and key points of cooperation in disease, treatment, rehabilitation and postoperative lifestyle, patiently answering patients' doubts, psychological guidance at the right time, preoperatively informing them that they have made adequate preparation for surgery to enhance their sense of security, and postoperatively working together with patients' families to enhance patients' confidence and self-control in fighting disease with suggestion and demonstration therapy; (2) preoperative gastrostomy tube and urinary catheter were not routinely placed, or left in place after anesthesia and removed after awakening; (3) preoperative intestinal preparation such as mechanical enema was not allowed; (4) atropine 0.5 mg + midazol 3 mg was injected intramuscularly before induction of anesthesia; (5) on the basis of routine general anesthesia care, the necessary warming measures were taken; (6) ostoperative drainage tube was not routinely left in place or removed 12 ~ 24 h after surgery; (7) postoperative pain pump was continuously pumped intravenously with long-acting local anesthetics and non-opioid analgesics was oraled for pain relief; (8) eating by mouth early after surgery, drinking water 6 h after surgery, transition to semi-liquid food 24 ~ 36 h after surgery; (9) postoperative 0 ~ 6 h bed activity, 6 ~ 24 h out of bed activity; (10) fluid control within 1,500 ml on the postoperative day.

Continuous care after discharge care: Before discharge, patients were given discharge instructions and introduction to the content of continuous care, and health knowledge manuals and postoperative assessment forms were issued, mainly including postoperative complications, mental, diet, sleep, exhaustion, urination, and defecation and precautions. A contact book was established and daily telephone follow-ups were made to assess patients' recovery and provide rehabilitation guidance, answering patients' questions, encouraging scientific exercise, and suggesting early return to work for those who recovered well.

### Observation Index

#### Care Effect

After care, if the patients' clinical symptoms disappeared and their conditions gradually recovered, it was regarded as efficient; if the patients' symptoms were obviously relieved and their conditions improved, it was regarded as effective; not meeting the above criteria was regarded as ineffective. Total effective number = total - ineffective number.

#### Surgical Stress Levels

The physiological stress levels and psychological stress levels of the two groups were compared 1 h before and 1 d after surgery.The former was assessed by enzyme-linked immunoassay (kit purchased from Shanghai Jianlai Biotechnology Co., Ltd.) for the detection of epinephrine and cortisol levels in fasting venous blood; the latter was assessed by the Hamilton anxiety scale (HAMA), which consisted of 14 items, with each item assigned a score of 0–4 and a total score of ≥7 being the presence of anxiety symptoms.

#### Postoperative Recovery

The time of first exhaust, first meal, first getting out of bed and hospitalization in both groups were recorded.

#### Complications

The number of complications such as incisional pain, infection, bile leak, and bleeding (including intra-abdominal bleeding and bleeding from the puncture incision) during care was counted in both groups.

#### SF-36 score

At the first follow-up visit after discharge, the SF-36 was used to assess the quality of life in both groups. It consisted of 8 dimensions, that was, physical functioning (PF), role physical (RP), bodily pain (BP), general health (GH), vitality (VT), social functioning (SF), mental health (MH), health transition (HT). Each dimension accounted for 100 points, and the score was proportional to the quality of life.

#### Care Satisfaction

At the first follow-up visit after discharge, the hospital's homemade Patient Satisfaction Questionnaire was used for assessment. It consisted of five entries, each of which was assigned a value of 1 ~ 3 points, with a total score of 12 ~ 15 as satisfied, 8 ~ 11 as average, and 5 ~ 7 as unsatisfied.

### Statistical Methods

SPSS 22.0 software was applied, and the measurement data were expressed as mean ± standard deviation and compared by *t*-test. Count data were expressed as ratio, and the χ^2^ test was used for comparison. *P* < 0.05 was considered statistically significant.

## Results

### Comparison of Care Effect Between the Two Groups

**Figure 1:**
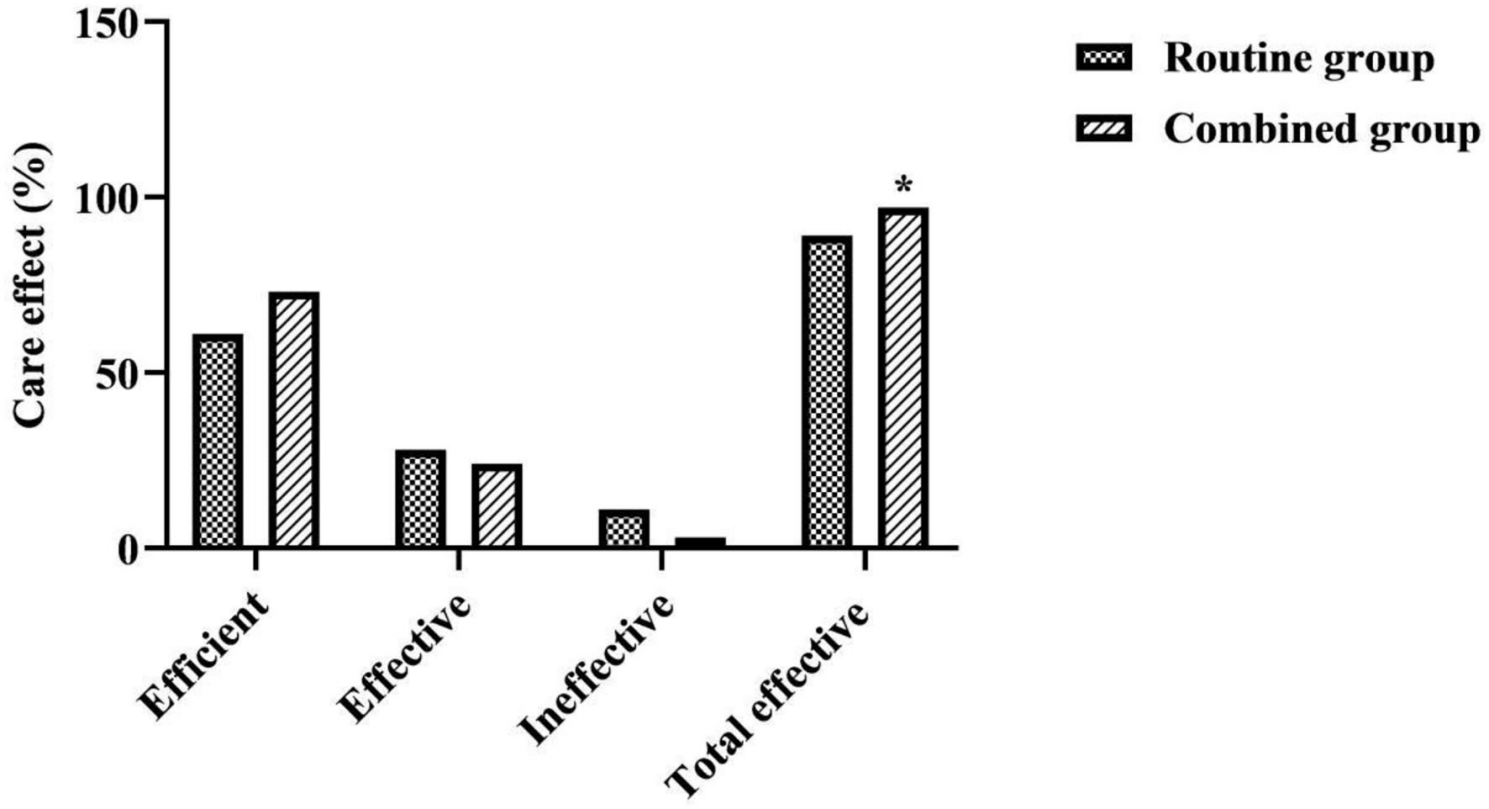
Comparison of care effect between the two groups. *was a comparison with the same item in the routine group, *P* < 0.05.

After care, the total efficiency of care in the combined group was better than that in the routine group (χ^2^ = 4.916, *P* = 0.027) (Figure 1).

### Comparison of Surgical Stress Levels Between the Two Groups

**Figure 2:**
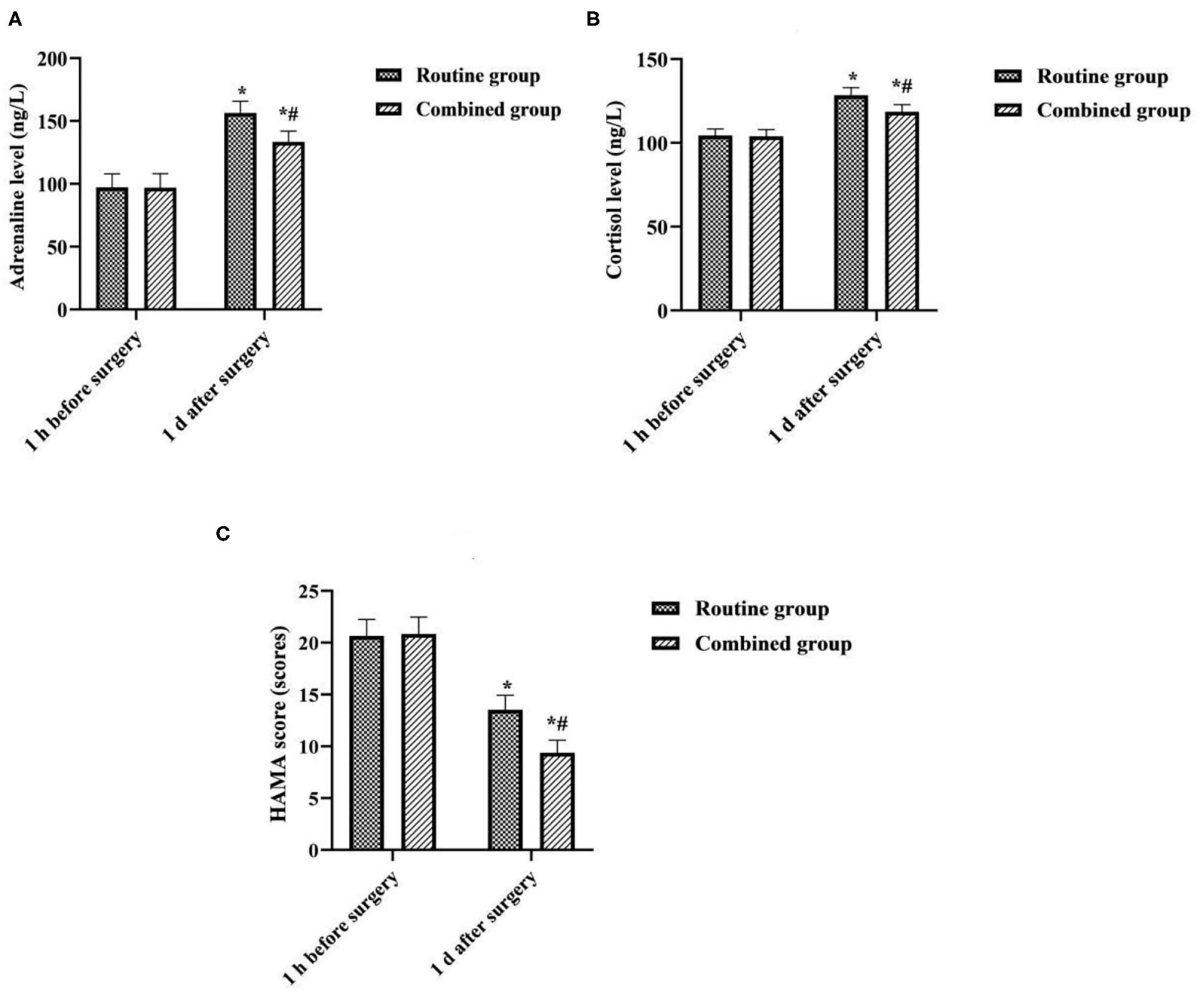
Comparison of surgical stress levels between the two groups. *was compared with the same group 1 h before surgery, *P* < 0.05; ^#^ was compared with the routine group 1 d after surgery, *P* < 0.05. **(A)** Adrenaline level. **(B)** Cortisol level. **(C)** HAMA score.

At 1 d after surgery, the levels of epinephrine and cortisol in both groups were significantly higher than those at 1 h before surgery, and the HAMA scores were significantly lower than those at 1 h before surgery, and the combined group was lower than the routine group (*P* < 0.05) (Figure 2).

### Comparison of Postoperative Recovery Between the Two Groups

**Figure 3:**
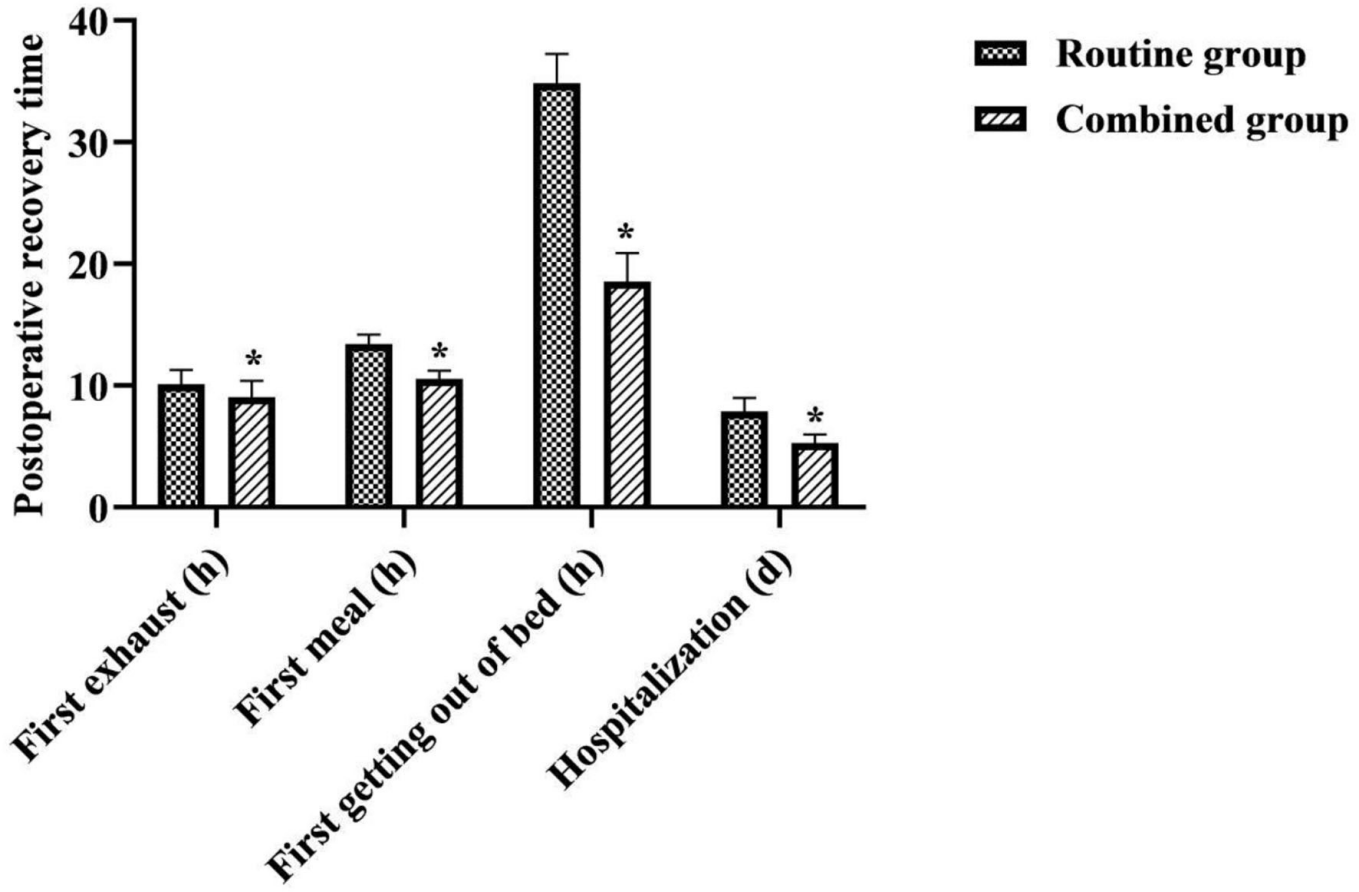
Comparison of postoperative recovery between the two groups. *was a comparison with the same item in the routine group, *P* < 0.05.

The time to first exhaustion, time to first meal, time to first getting out of bed, and time to hospitalization were shorter in the combined group than in the routine group (*P* < 0.05) (Figure 3).

### Comparison of Complication Rates Between the Two Groups

**Figure 4:**
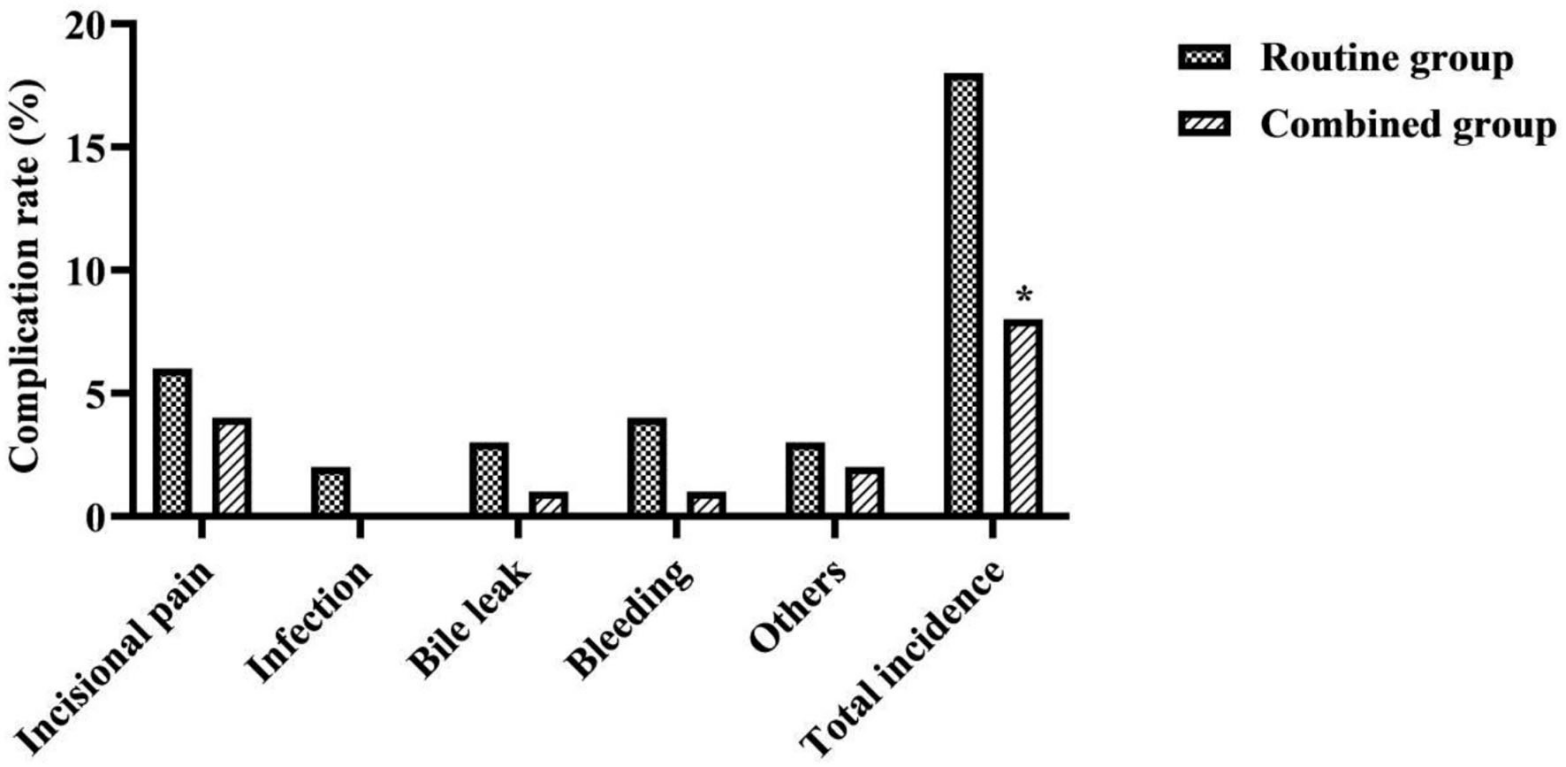
Comparison of complication rates between the two groups. *was a comparison with the same item in the routine group, *P* < 0.05.

The overall complication rate in the combined group was lower than that in the routine group (χ^2^ = 4.421, *P* = 0.036) (Figure 4).

### Comparison of SF-36 Scores Between the Two Groups

**Figure 5:**
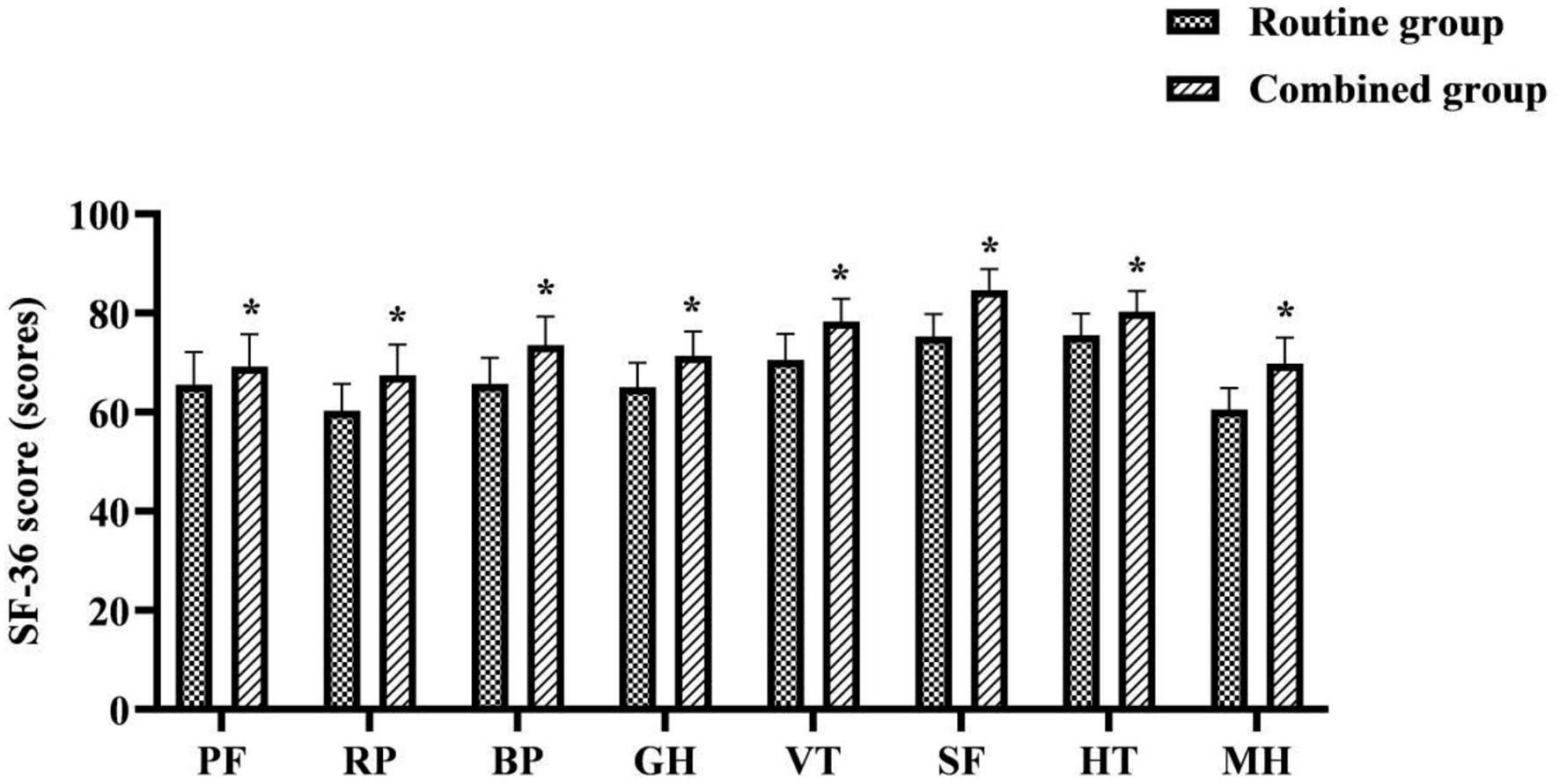
Comparison of SF-36 scores between the two groups. *was a comparison with the same item in the routine group, *P* < 0.05.

The each item of SF-36 scores after discharge were higher in the combined group than in the routine group (*P* < 0.05) (Figure 5).

### Comparison of Care Satisfaction Between the Two Groups

**Figure 6:**
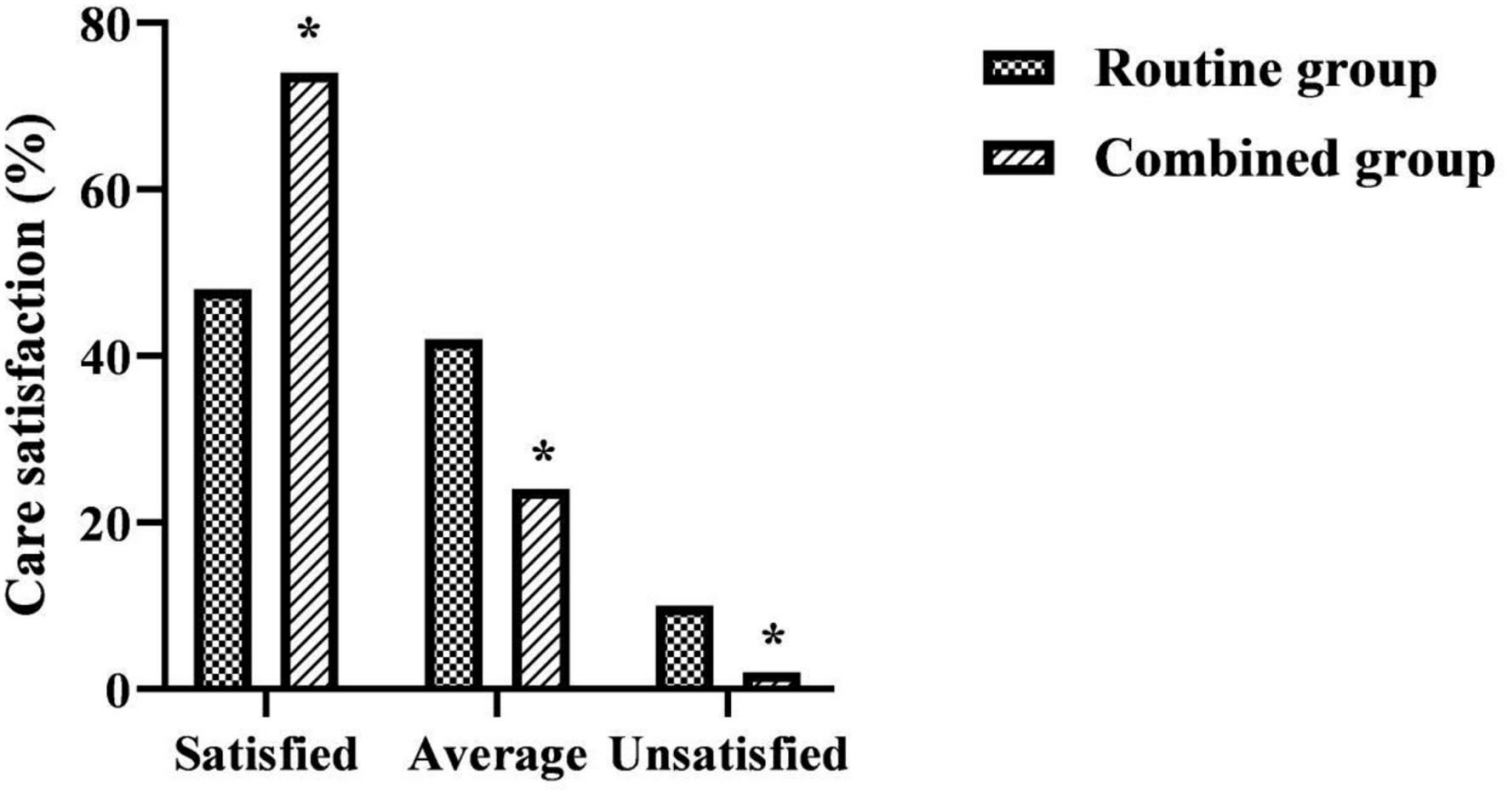
Comparison of care satisfaction between the two groups. *was a comparison with the same item in the routine group, *P* < 0.05.

The total satisfaction with care was higher in the combined group than in the routine group (χ^2^ = 8.416, *P* = 0.015 (Figure 6).

## Discussion

Gallbladder disease is one of the common diseases that threaten human health. In China, the incidence of gallbladder stones is 10–15% and the incidence of cholecystitis is 28%. The main clinical method of treatment is surgical excision. At present, the LC technique in China is relatively mature, and its damage to abdominal muscles, fascia, nerves and blood vessels is small, and patients can have no obvious external trauma after surgery, leaving only one to four small incisions, which facilitates their postoperative recovery (13, 14). However, the somatic trauma, physiological stress, metabolic changes, and the resulting painful stimuli and anxiety caused by surgery can affect several systems of patients, including neurological, endocrine, and circulatory (15, 16). This interferes with the outcome of surgical treatment for LC patients and delays their postoperative physical and psychological recovery.

Care interventions are an important part of clinical care. In this study, FTS care combined with continuous care after discharge was applied to LC patients, and the results showed that the total efficiency of care in the combined group was better than that in the routine group (*P* < 0.05). At 1 d after surgery, the levels of epinephrine and cortisol in both groups were significantly higher than those at 1 h before surgery, and the HAMA scores were significantly lower than those at 1 h before surgery, and the combined group was lower than the routine group (*P* < 0.05). The time to first exhaustion, time to first meal, time to first getting out of bed, and time to hospitalization were shorter in the combined group than in the routine group (*P* < 0.05). The overall complication rate in the combined group was lower than that in the routine group (*P* < 0.05). Those suggests that providing comprehensive, optimized, and compact care care to LC perioperative patients is very beneficial to ensure successful surgery, reduce surgical stress, and accelerate patient recovery.

In the conventional care model: LC patients need to undergo a longer period of preoperative fasting and no drinking, which can lead to irritability, thirst, hunger, dehydration, hypoglycemia, and hypovolemia, increasing body consumption and affecting postoperative tissue repair and wound healing (17). Routine preoperative indwelling of gastric and urinary catheters can cause discomfort and limited early postoperative activities, and increase the risk of respiratory and urinary tract infections (18). Preoperative bowel preparation such as receiving mechanical enemas predisposes patients to preoperative dehydration, increasing the risk of hypotension during anesthesia, and can also cause intestinal edema, increasing the chance of postoperative intestinal paralysis and abdominal infection (19). During intraoperative care, the patient's stressful response to hypothermia can impair coagulation mechanisms, the cardiovascular system, and leukocyte function (20, 21). Retained drains can cause pain, limited mobility, or retrograde infection in patients. The use of opioid analgesics and prolonged bed rest can affect the recovery of postoperative gastrointestinal function (22). Longer postoperative fasting can lead to malnutrition in patients and affect wound healing (23). In terms of fluid control, due to prolonged fasting and abstinence from food and drink, in order to maintain water and electrolyte balance, patients are often clinically over-infused, triggering pulmonary edema or intestinal edema, aggravating postoperative intestinal paralysis and affecting intestinal motility (24). In addition, surgery, as a major negative life event, can lead to a series of physiological and psychological behavioral reactions such as tension and fear, which in turn can lead to adverse emotions such as anxiety, affecting the surgical outcome and exacerbating the damage caused by surgical stress on the organism, which is detrimental to the patient's postoperative recovery (25).

In contrast, in the FTS care model: the short duration of patient fasting and drinking helps to relieve patients' preoperative thirst and hunger, avoid the development of postoperative insulin resistance, and reduce the postoperative stress response, helping to maintain a good metabolic status after surgery (26, 27). Not routinely placing gastric tubes, urinary catheters and drainage tubes helps to avoid uncomfortable irritation, stress and related complications caused by medical measures. The necessary intraoperative insulation measures can reduce the stress damage caused by hypothermia to the organism. In terms of postoperative analgesia, the combination of pain pumps and opioid analgesics has been shown to be effective in relieving pain stress caused by surgical trauma and creating conditions for the patient to get out of bed early (28). Early postoperative trans oral feeding helps to promote intestinal peristalsis, maintain intestinal mucosal function, shorten postoperative anal venting time, and to a certain extent reduce the amount of postoperative fluids, which in turn helps to prevent hypoxemia and pulmonary edema and promote recovery of gastrointestinal function. Postoperative getting out of bed early promotes the recovery of gastrointestinal, bladder and respiratory system functions, which in turn helps to reduce the occurrence of abdominal distention, urinary retention, pulmonary complications and deep vein thrombosis. In addition, a variety of preoperative health education and psychological care can effectively reduce patients' doubts, tension and anxiety, and can effectively relieve patients' postoperative pain. The above care measures will ultimately help to reduce physical and psychological stress and postoperative complications, as well as increase patients' confidence and compliance with treatment, and therefore will be beneficial in accelerating their recovery process.

Surgery, as an invasive treatment measure, inevitably leads to a series of pathological and physiological changes in the body while achieving therapeutic results, resulting in varying degrees of reduced quality of life for patients (29). In the results of this study, the each item of SF-36 scores after discharge were higher in the combined group than in the routine group (*P* < 0.05). The total satisfaction with care was higher in the combined group than in the routine group (*P* < 0.05). This suggests that the use of FTS care combined with continuous care after discharge for LC patients contributes to their quality of life and satisfaction. As a minimally invasive treatment, patients can be discharged from the hospital to continue treatment at home in a short time after LC. However, under the routine care model, it is difficult for patients to receive additional medical support after discharge from the hospital. The implementation of continuous care after discharge in this study is a model of care that provides continuous targeted rehabilitation guidance for a period of time after the patient is discharged from the hospital. Its daily communication with patients in the form of telephone follow-up, medical and nursing staff can keep abreast of patients' recovery, and can encourage patients to exercise and exercise, supervise their healthy diet and ease their psychological problems through the telephone. This will help the patient to face the post-operative rehabilitation with a positive attitude, which will eventually help the patient to return to normal life or even early return to work, etc. as soon as possible. While meeting the care needs of patients in the rehabilitation stage, it also enhances communication between nurses and patients and promotes care-patient friendship, which is therefore very beneficial to improving patients' quality of life and satisfaction with care effect after discharge.

## Conclusion

The implementation of FTS care combined with continuous care after discharge in LC patients is ideal, which can significantly reduce the level of surgical stress, accelerate the recovery process and reduce the occurrence of complications, and improve the postoperative quality of life of patients significantly and with high satisfaction, which is worthy of application.

## Data Availability Statement

The original contributions presented in the study are included in the article/supplementary material, further inquiries can be directed to the corresponding author/s.

## Ethics Statement

The studies involving human participants were reviewed and approved by the Ethics Committee of the First People's Hospital of Lianyungang. The patients/participants provided their written informed consent to participate in this study.

## Author Contributions

All authors listed have made a substantial, direct, and intellectual contribution to the work and approved it for publication.

## Conflict of Interest

The authors declare that the research was conducted in the absence of any commercial or financial relationships that could be construed as a potential conflict of interest.

## Publisher's Note

All claims expressed in this article are solely those of the authors and do not necessarily represent those of their affiliated organizations, or those of the publisher, the editors and the reviewers. Any product that may be evaluated in this article, or claim that may be made by its manufacturer, is not guaranteed or endorsed by the publisher.
